# Advances in Isofuranodiene Extraction from *Smyrnium olusatrum* L.: Supercritical Carbon Dioxide Extraction

**DOI:** 10.3390/plants15071099

**Published:** 2026-04-03

**Authors:** Eleonora Spinozzi, Giada Trebaiocchi, Riccardo Petrelli, Francesco Di Monaco, Marco Cespi, Filippo Maggi

**Affiliations:** Chemistry Interdisciplinary Project (ChIP), School of Pharmacy, University of Camerino, Via Madonna Delle Carceri, 62032 Camerino, Italy; giada.trebaiocchi@unicam.it (G.T.); riccardo.petrelli@unicam.it (R.P.); francesco.dimonaco@studenti.unicam.it (F.D.M.); marco.cespi@unicam.it (M.C.); filippo.maggi@unicam.it (F.M.)

**Keywords:** isofuranodiene, *Smyrnium olusatrum* L., thermal degradation, supercritical CO_2_

## Abstract

Supercritical CO_2_ (S-CO_2_) extraction is one of the most employed techniques for the extraction of bioactive compounds for its safety, effectiveness, cost-efficiency, and good environmental compliance. *Smyrnium olusatrum* L. (Apiaceae) is an aromatic plant of great interest due to its potential applications in pharmaceutical, agrochemical, and oleochemical fields. Its bioactivity is caused by furanosesquiterpenes, mainly represented by isofuranodiene (IFD). The extraction of this compound is usually achieved through Soxhlet or hydrodistillation. However, the latter usually leads to the thermal Cope rearrangement of IFD into its isomer curzerene, resulting in low recovery. This study reported for the first time the optimization of S-CO_2_ extraction of IFD from *S. olusatrum* schizocarps. Pressure (MPa), extraction time (min), and static mode (%) were varied while the temperature was maintained at 45 °C to avoid IFD thermal degradation. The optimized process (50 MPa, 60 min, 25% static mode) provided an extraction yield and an IFD recovery of 8.50 and 0.94% and avoided the thermal degradation of the compound. This study demonstrated that S-CO_2_ extraction is a valuable alternative to conventional hydrodistillation (extraction yield and IFD recovery of 2.64 and 0.77%) and Soxhlet (extraction yield and IFD recovery of 9.49 and 0.85%) to recover IFD from *S. olusatrum*.

## 1. Introduction

*Smyrnium olusatrum* L., commonly known with the name of Alexanders, is a biennial herb belonging to the Apiaceae family. Although nowadays it is considered a neglected species, it was largely employed until the Middle Ages for food purposes as well as in traditional medicine. This plant has gained an increasing research interest in recent years due to the presence of furanosesquiterpenes, mainly represented by isofuranodiene (IFD) [1,2,3,4]. The latter has been accounted for as the main responsibility for the plant’s biological activities, such as being anticancer, hepatoprotective, antimicrobial, and insecticidal to name a few [1]. Normally, IFD is obtained from the plant through conventional hydrodistillation [4,5], but this technique presents some weaknesses mainly linked to the application of high temperatures on the plant material. Indeed, IFD is a thermosensitive compound that undergoes a pericyclic reaction when heated, which is called Cope rearrangement. This process leads to the consequent formation of its isomer curzerene lowering the recovery of IFD [6,7,8]. This feature, together with the high energy and time consumption, makes hydrodistillation a constrained technique for the recovery of this compound. An alternative strategy for IFD extraction could be represented by conventional solid–liquid extraction, but this approach presents some limitations especially when not optimized. Indeed, it often leads to high time and energy consumption accompanied by the employment of high volumes of organic solvents. The latter are not always safe for the operator and for the environment, in addition to not always being industrially employable [9]. Furthermore, the resulting extracts are often unsafe due to solvent residues or degraded compounds formed by extreme extraction conditions [9,10]. In this context, supercritical fluid extraction is a well-known alternative to conventional extraction processes [11,12], with carbon dioxide (CO_2_) as the most utilized solvent [11,13,14,15,16]. This technique has been applied to diverse plant matrices, often leading to high bioactive recoveries in many cases [11]. Given the potential applications of IFD in pharmaceutical, nutraceutical, and agrochemical fields, this work aimed for the first time to optimize its extraction from *S. olusatrum* fruits by using S-CO_2_. This study aimed to demonstrate the added value of S-CO_2_ for IFD extraction as a valid alternative to conventional hydrodistillation by achieving similar or improved extraction efficiency, reducing extraction times, and avoiding IFD thermal degradation.

## 2. Results

### 2.1. HPLC-DAD Method Validation

The method was validated in terms of linearity, repeatability, limit of detection (LOD) and limit of quantification (LOQ). The method linearity was determined by analyzing different concentrations of IFD and curzerene and determining the coefficient of determination (R^2^) (0.993) (Figure S1, Supplementary Material). The repeatability was verified by determining the relative standard deviation (%RSD) between consecutive analyses (n = 5) carried out in the same day (intraday reproducibility) (0.87 and 1.27% for IFD and curzerene, respectively) and during 5 consecutive days (interday reproducibility) (0.98 and 1.56% for IFD and curzerene, respectively). The LOD and LOQ were experimentally calculated by injecting low concentrations of the IFD and curzerene standard solutions and measuring the signal-to-noise (S/N) ratio and they were 0.045 and 0.15 μg/mL for IFD and 0.91 and 3.01 μg/mL for curzerene, respectively.

### 2.2. Supercritical CO_2_ Extraction (S-CO_2_ Extraction)

#### 2.2.1. Preliminary Screening

The aim of the preliminary screening was to evaluate if the identified predictors for the S-CO_2_ extraction process, namely pressure, time, and static mode (%), affected the extraction process in terms of extraction yield (%) and IFD recovery (%), as reported in Section 4.5 (Table 1).

**Figure 1:**
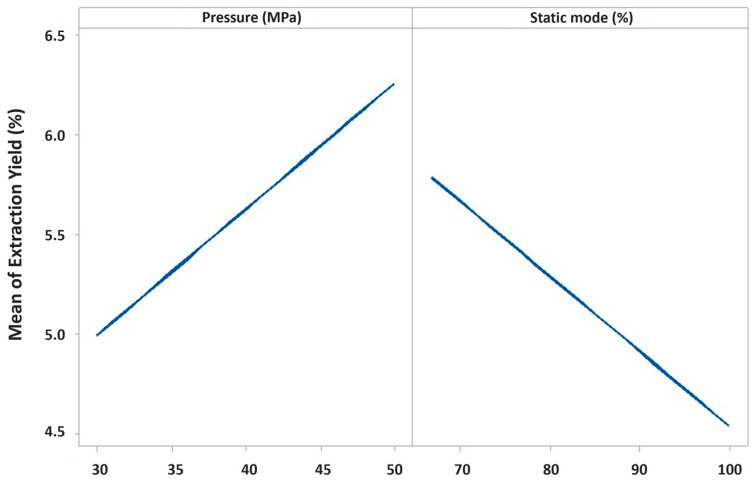
Mean effect of the predictors pressure (MPa) and static mode (%) on extraction yield (%).

Table 1 reports the results obtained in the preliminary screening. In regard to the extraction yield, it ranged from 1.71 (trials P2 and P7) to 2.30% (trial P5). Concerning the IFD recovery (%), it ranged from 0.57 (trial P1) to 0.78% (trial P3). The above-mentioned results were examined through regression analysis. In detail, the evaluation of the goodness of fit parameters revealed good descriptive and predictive capacities both in terms of extraction yield (R^2^_adj_ 0.732; R^2^_pred_ 0.626) and IFD recovery (R^2^_adj_ 0.791; R^2^_pred_ 0.739). Additionally, the regression was significant. Details of the regression analysis for extraction yield and IFD recovery are reported in Figures S2 and S3 of the Supplementary Material, respectively. The regression analysis revealed that the S-CO_2_ extraction predictors mainly influencing the extraction process were pressure and static mode (%) for the extraction yield and exclusively static mode (%) for the IFD recovery (%). In detail, both predictors influenced the extraction yield with comparable intensity, although in opposite directions (Figure 1).

**Figure 2:**
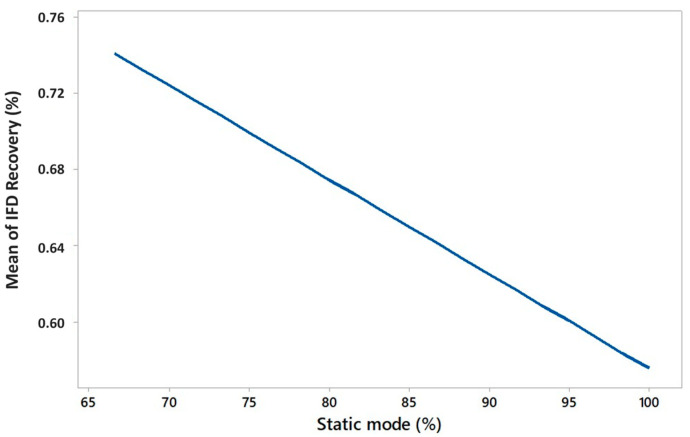
Mean effect of static mode (%) on IFD recovery (%) during S-CO_2_ extraction.

Specifically, an increase in pressure led to a higher yield, whereas an increase in static mode (%) resulted in a decrease in yield. The combined effect of pressure and static mode (%) on the extraction yield can also be visualized using the contour plot as reported in Figure S4 of the Supplementary Material. Regarding the IFD recovery (%), it was affected exclusively by the static mode (%). Low values of static mode (%) determined a higher IFD recovery (%) (Figure 2). For completeness, it must be highlighted that main effect plots (Figure 1 and Figure 2) and response surface visualizations (Figure S4) represent qualitative graphical aids to facilitate interpretation of regression results rather that quantitative or prediction tools.

#### 2.2.2. Optimization

Following the results of the regression analysis on the preliminary trials, the optimization was conducted only varying two predictors, namely pressure and static mode (%). Regarding the pressure, it was not further increased due to the safety limitations of the instrument (50 MPa). For this reason, two constant values of pressures were set for the optimization trials, namely 30 and 50 MPa, varying the static mode (%). Both extraction yield and IFD recovery (%) were evaluated for each optimization trials (Table 2).

**Figure 3:**
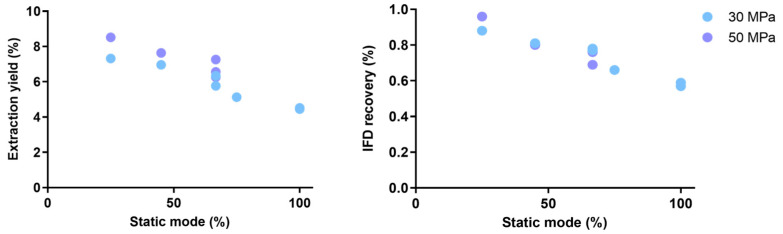
General trend of the effect of the static mode (%) at the two different pressures (30 and 50 MPa).

The results of the yield and IFD recovery (%) are reported in Figure 3. A general trend for the two considered answers can be observed, in line with the results of preliminary screening and confirming that the static mode (%) is the most influencing predictor both for the extraction yield (%) and the IFD recovery (%) from the plant matrix.

More in detail, the higher is the static mode (%) during the process, the lower is the recovery of IFD (%). Concerning the pressure, it showed a modest effect on the extraction yield that can be observed mainly at low values of static mode (%), while it is confirmed that it does not affect the IFD extraction (Figure 3). Additionally, the graphical results do not suggest meaningful interaction patterns within the investigated experimental region. Therefore, additional experiment runs or more structured modelling was not pursued.

For the above-mentioned reasons, the optimized conditions were those of the trial O12 (temperature of 45 °C, pressure of 50 MPa, 25% of static mode). For a more robust evaluation, the extraction following the conditions of trial O12 was repeated in triplicate generating the following results: extraction yield of 8.50 ± 0.03% and IFD recovery of 0.94 ± 0.03%.

### 2.3. Comparison with Conventional Extraction Techniques

To evaluate the efficiency of the S-CO_2_ extraction process in terms of IFD extracted from the matrix, the optimized conditions were compared to the conventional extraction techniques used for IFD recovery, namely hydrodistillation and Soxhlet. The extraction yield obtained with hydrodistillation resulted in 2.64%, while the IFD recovered from the fruits was 0.77%. Regarding the Soxhlet extraction, the extraction yield and the IFD recovered from the fruits resulted in 9.49 and 0.85%, respectively.

**Figure 4:**
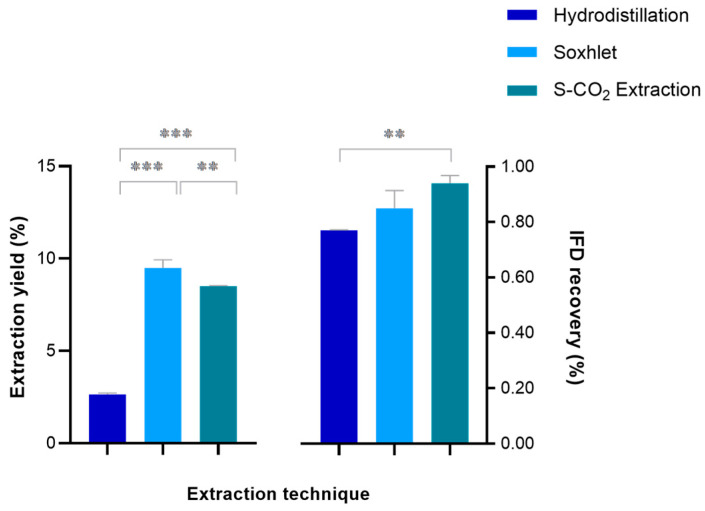
Mean values (%) and standard deviation (n = 3) of extraction yield (%) and IFD recovery (%) of the hydrodistillation, Soxhlet, and supercritical CO_2_ extraction (S-CO_2_ extraction). The asterisks represent the significance of Tukey’s test according to the following: *** *p* < 0.001, and ** 0.01 < *p* < 0.001.

On the other hand, the extraction yield (%) and the IFD recovery (%) obtained with the S-CO_2_ extraction were 8.50 and 0.94%, respectively. The extraction yield among the three different techniques results were statistically different when tested with the Anova followed by Tukey’s test (Figure 4).

Soxhlet appeared comparable to the S-CO_2_ extraction although a proper evaluation cannot be formulated due the high variability of the results.

#### Quality Assessment of the Essential Oil/Extracts

**Figure 5:**
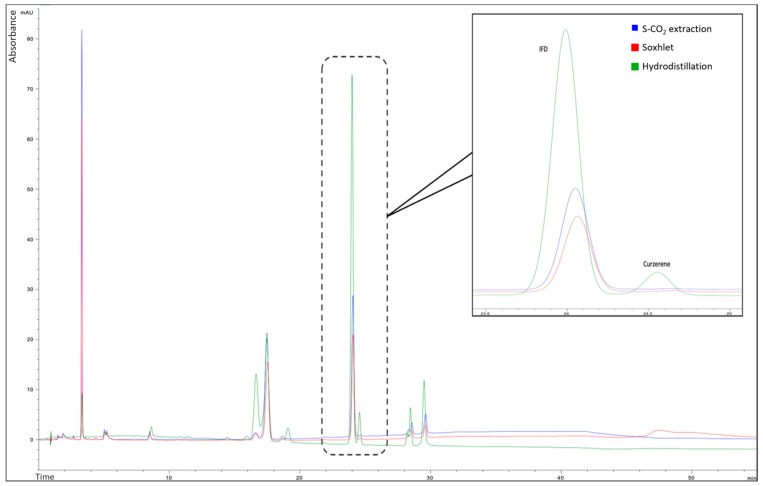
HPLC-DAD chromatograms of the essential oil/extracts obtained from hydrodistillation, Soxhlet, and S-CO_2_ extraction. Different colours are related to the extraction technique: green, hydrodistillation; red, Soxhlet; blue, S-CO_2_.

All the techniques results were effective in terms of the IFD extracted (%). However, the optimization of the S-CO_2_ extraction process was carried out aiming to avoid the thermal rearrangement of IFD into curzerene, usually happening at high temperatures. For this reason, the essential oil and the S-CO_2_ and Soxhlet extracts were analyzed in HPLC-DAD to assess the potential presence of curzerene. As reported in Figure 5, the hydrodistillation process favoured the thermal degradation of IFD and the consequent formation of curzerene (4.04 ± 0.05%), while S-CO_2_ extraction and Soxhlet avoided this phenomenon. This may be due to partial Cope rearrangement of IFD into curzerene following strong extraction conditions (e.g., high temperatures, hydrolysis, oxidation) occurring during the hydrodistillation of the fruits. On the other hand, it can be stated that both S-CO_2_ and Soxhlet extractions are effective in preserving IFD from thermal conversion.

## 3. Discussion

IFD was previously extracted from *S. olusatrum* fruits by using S-CO_2_ only by Marongiu et al. [17], who performed an S-CO_2_ extraction with extraction yields ranging from 1.4 to 2.2%. In this case, the extraction was performed with a different equipment at 1.5 MPa and at 10 °C and an optimization of the process was not performed.

So, this is the first study reporting the use of S-CO_2_ extraction to improve the recovery of IFD. The optimization of the S-CO_2_ extraction process depends significantly on the employed matrix and its related analytes. Moreover, the predictors such as time, pressure, static mode (%), and temperature can have results of varying importance. In this work, the static mode (%) was the most influencing predictor both for the extraction yield (%) and the IFD recovery (%) from the plant matrix. This result is justified by the fact that a higher % of dynamic mode allows the continuous refresh of the CO_2_ inside the extraction chamber during the whole process, thus enhancing the extraction efficiency. This result aligns with that obtained for the recovery of cannabinoids from hemp biomass using the same equipment [18]. Indeed, static mode (%) also influenced the extraction yield, with higher values reached with a lower % of static mode. In the above-mentioned study, pressure resulted in another predictor influencing the extraction yield, with higher values obtained with higher pressures [18]. The result reported by Spinozzi et al. [18] partially differs from that obtained in our study, in which the influence of pressure on the extraction process was not as significant as the % of static mode. This difference could be potentially ascribed to the different matrix employed in the study, which could store its bioactive compound in different anatomical structures than hemp. Concerning the static/dynamic mode, other works with the same instrument also reported the employment of this mode to obtain high recovery yields of bioactive compounds. This was the case of the work of Pompei et al. in which a static mode (%) of 40% was adopted for the extraction of bioactives from Lacrima di Morro d’Alba grape pomace and Ascolana Tenera olive leaves [19].

The efficiency of the S-CO_2_ extraction process was evaluated by comparison to conventional extraction techniques used for IFD recovery, namely hydrodistillation and Soxhlet. The extraction yield obtained with hydrodistillation (2.64%) highly differs from that previously published (0.2 to 0.3%) [4] and this is probably linked to the lack of a preventive shredding of the fruits before the extraction. Regarding the Soxhlet extraction, *S. olusatrum* fruits have been previously extracted by stirring with *n*-hexane at room temperature with an extraction yield of 5.7% [4]. The lower value of yield obtained in this case with respect to our data (8.50%) could be linked to the lack of the preventive shredding or to the higher extraction efficiency of the Soxhlet technique with respect to a normal stirring extraction. Nevertheless, the differences detected among the three techniques in terms of extraction yield are not surprising since the processes and the obtained products are completely different: i.e., an essential oil (only lipophilic volatile compounds) from the hydrodistillation and two lipophilic extracts (containing both volatile and not volatile compounds) from the S-CO_2_ and Soxhlet extractions.

## 4. Materials and Methods

### 4.1. Chemicals and Reagents

IFD employed as a reference standard was obtained through a crystallization procedure, as reported in Section 2.3. HPLC-grade acetonitrile was purchased from Merck (Darmstadt, Germany), while deionized water (>18 MΩ cm resistivity) was further purified using a Milli-Q SP Reagent Water System (Millipore, Bedford, MA, USA). Before the analysis, all solvents and solutions were filtered through a 0.2 µM polyamide filter from Sartorius Stedim (Goettingen, Germany).

### 4.2. Plant Material

Ripe fruits (dry schizocarps) of *S. olusatrum* were collected in Camerino, central Italy (43°07′52″ N 13°03′59″ E 589 m), in July 2024. The Herbarium specimen was identified by Prof. Filippo Maggi and deposited at the Herbarium Universitatis Camerinensis, c/o School of Biosciences and Veterinary Medicine of the University of Camerino, Italy, under the code CAME 29339.

### 4.3. Isofuranodiene Purification and Curzerene Synthesis

IFD was isolated from the essential oil of *S. olusatrum* schizocarps following the methodology previously reported [19]. In detail, the dry fruits were shredded and then subjected to hydrodistillation, as reported in Section 4.6.1. Then, the obtained essential oil was diluted in *n*-hexane (purity >98%, Merck, Darmstadt, Germany) (in a 1:10 *w*/*v* ratio) and stored at −20 °C to allow the precipitation of IFD crystals. The precipitation yield (70%) was consistent with data from the literature [20]. The latter were then subjected to filtration and to desiccation prior to chemical characterization. Curzerene was synthesised according to the procedure of Baldovini et al. [21] using IFD as the starting material, and the reaction yield (quantitative) was consistent with that which has been previously reported [21]. The IFD and curzerene structures and purity were confirmed through NMR analysis (^1^H NMR and ^13^C NMR—Bruker Avance III 500 MHz spectrometer, Billerica, MA, USA), after solubilization in deuterated chloroform, and they were linear with those previously reported [20,21].

### 4.4. HPLC-DAD Analysis

#### 4.4.1. Stock Solutions Preparation

The IFD and curzerene stock solutions (1000 µg/mL) were prepared by diluting the compounds in HPLC-grade acetonitrile. The essential oil obtained from hydrodistillation was diluted in HPLC-grade acetonitrile (1000 µg/mL). The extracts deriving from the S-CO_2_ extraction were diluted in HPLC-grade acetonitrile (5000 µg/mL). The obtained solution was diluted 1:5 with the mobile phase chosen for HPLC-DAD analysis (H_2_O:MeCN, 60:40% *v*/*v*). All the solutions were vigorously mixed with a Vortex device for 1 min and then sonicated for 3 min in an ultrasound bath. Finally, they were filtered with a 0.2 μm filter and stored at −20 °C before analysis.

#### 4.4.2. Analytical Conditions

The analysis was conducted employing an HPLC Agilent 1100 series (Agilent Technologies, Santa Clara, CA, USA) equipped of a binary solvent pump, an autosampler, and a Photodiode Array Detector (DAD), controlled by a ChemStation (Agilent, v.01.03). The separation of the analytes was achieved by employing a Kinetex PFP 100 A column (100 × 4.6 mm i.d., 2.6 μm), purchased from Phenomenex (Torrance, CA, USA) and operating at 40 °C. The analytical conditions followed those previously reported by Maggi et al. [4]. Specifically, a gradient elution consisting of water (A) and acetonitrile (B) was adopted (flow of 1.0 mL/min): 0–15 min, 40% B; 15–30 min, 60% B; 30–40 min, 60% B. The injection volume was 1 µL. For quantitative analysis, the wavelength monitored was 230 nm.

#### 4.4.3. Method Validation

Each solution was analyzed in duplicate. The method was validated in terms of linearity, repeatability, limit of detection (LOD), and limit of quantification (LOQ). The method linearity was determined by analyzing different concentrations of IFD (1800 to 1 μg/mL) and curzerene (1111.5 to 0.95 μg/mL) and determining the coefficient of determination (R^2^). The repeatability was verified by determining the relative standard deviation (%RSD) between consecutive analyses (n = 5) carried out in the same day (intraday reproducibility) and during 5 consecutive days (interday reproducibility). The LOD and LOQ were experimentally calculated by injecting low concentrations of the IFD and curzerene standard solutions and measuring the signal-to-noise (S/N) ratio. A concentration giving a S/N ratio (height of peak/height of noise) of three was assigned to LOD while that of ten was assigned to LOQ.

### 4.5. Supercritical CO_2_ Extraction (S-CO_2_)

**Figure 6:**
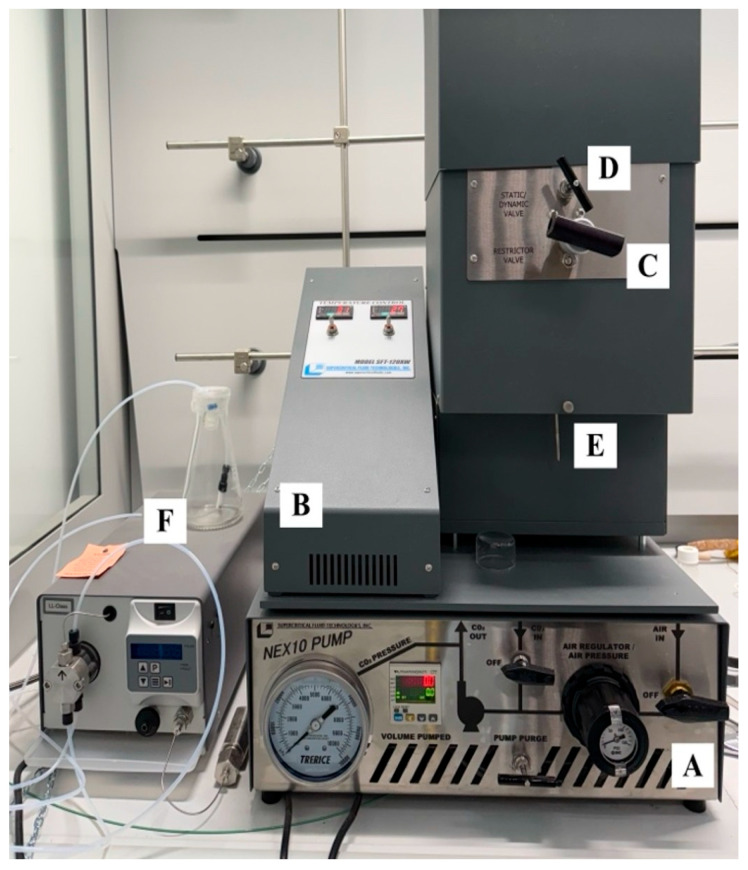
Components of the supercritical CO_2_ (S-CO_2_) extractor SFT-120XW: SFT-Nex10 pump regulating the flow of CO_2_ (A); co-solvent pump (B); temperature setting system (C); static/dynamic valve (D); restriction valve (E); collection point (F).

The S-CO_2_ extraction was conducted using an SFT-120XW Supercritical Fluid Extractor (Supercritical Fluid Technologies, Inc., Newark, DE, USA) (Figure 6) equipped of an extraction chamber of 100 mL that can operate at pressures up to 10.000 psi (69 MPa) and at temperatures up to 200 °C. The CO_2_ is supplied inside of the instrument by a SFT-Nex10 pump (Supercritical Fluid Technologies, Inc., Newark, DE, USA) (Figure 6). The latter is a high performance, pneumatically driven piston pump which rapidly compresses liquid CO_2_ from the tank pressure (750–900 psi) up to the pressures required for the S-CO_2_ extraction. The S-CO_2_ extractor is furnished with two manual valves: a static/dynamic valve and a restriction valve (Figure 6). The static/dynamic valve regulates the flow of CO_2_ inside the extraction chamber: in the static mode, the plant material is in static contact with the CO_2_, while in the dynamic mode, the CO_2_ flows (10 mL/min) continuously through the extraction chamber and inside the plant material, allowing a continuous CO_2_ renewal. At the end of both of the extraction modes, the restriction valve depressurizes the CO_2_ and the obtained extract from supercritical pressure to atmospheric pressure, allowing their flow in the collection point (Figure 6). The instrument allows the employment of a co-solvent to extract polar compounds through a specific pump. Each S-CO_2_ extraction was performed on *S. olusatrum* dry fruits (10 g) preventively shredded and then placed into a specific extraction filter. After each S-CO_2_ extraction, the extract was collected into specific EPA glass vials and then stored at 4 °C prior to chemical analysis.

Each S-CO_2_ extraction trial was evaluated in terms of extraction yield (% dry weight; DW) and IFD extracted (%), hereafter designated as ‘responses’.

(1) $$extraction\;yield\left(\%DW\right)=\frac{weight\;of\;extract\;(g)}{weight\;of\;fruits\;(g)}\times 100$$

(2) $$IFD\;extracted\;(\%)=\frac{IFD\;extracted\;(g)}{weight\;of\;fruits\;(g)}\times 100$$

The IFD extracted (%) was determined quantifying it through HPLC-DAD analysis, and details are reported in Section 4.4.

#### 4.5.1. Preliminary Screening

The critical parameters to be studied for the S-CO_2_ extraction of IFD from *S. olusatrum* were selected considering the previous results from our recent work [21] carried out using the same equipment on a different plant matrix. These parameters, specifically pressure, time, and static mode (%) (% of static time with respect to the total extraction time), hereafter denominated “predictor”, were here chosen to conduct the preliminary trials. The choice of using a static/dynamic mode was driven by the possibility of further enhancing IFD extraction efficiency, thanks to the continuous flow of CO_2_ inside of the extraction chamber. The values of static mode (%) were chosen to find the optimal compromise between extraction efficiency and CO_2_ consumption. The use of a co-solvent was excluded since IFD is a non-polar compound that can be easily extracted by using only CO_2_. Regarding the temperature, it has been maintained at 45 °C to avoid the Cope rearrangement of IFD. Details on the choice of this temperature are reported in the Supplementary Material (Section S2). The extraction time was set at 45, 60, and 90 min. The preliminary trials are reported in Table 3. Since limited initial knowledge of the plant matrix extraction with CO_2_ was available, preliminary trials were conducted as reported in Table 3 to understand how the variables could potentially influence the system.

#### 4.5.2. Regression Analysis for the Preliminary Screening Trials

The results of the preliminary tests were analyzed by multilinear regression using a linear model:

(3) $$y=\beta_{0}+\sum_{i=1}^{n}\beta_{i}\cdot x_{i}$$

where *y* is the response, *β*_0_ is the model constant and *β_i_* is the coefficient corresponding to the variables *x_i_* (linear terms). The obtained full quadratic models were subjected to a reduction procedure to improve the accuracy of the estimated coefficients for the selected variables, minimize the mean quadratic error and generally respect the principle of parsimony [22,23]. Model reduction was performed by stepwise regression in backward elimination mode and all the identified models were compared by means of the adjusted multiple determination coefficient (R^2^_adj_), the predicted multiple determination coefficient (R^2^_pred_), and Mallows’ Cp statistic. The regression process was evaluated by means of variance analysis of the model (ANOVA) and residual analysis. Both model fitting and regression analysis were performed using Minitab 18 statistical software. The results of multiple regression analyses are reported using the main effect graphs and/or contour plots.

#### 4.5.3. Optimization

According to the preliminary regression analysis, it was decided to optimize the S-CO_2_ extraction exclusively in relation to the predictors static mode (%) and pressure. It shall be specified that, also based on the results obtained from the analysis of the preliminary trials, there were divergent results for pressure regarding yield and concentration of IFD. After the results from the preliminary screening, it was decided to carry out further experimental tests (four optimization tests) in which the static mode (%) was lowered at both high pressures (50 MPa) and low pressures (30 MPa). The conditions used for optimization tests are shown in Table 4.

### 4.6. Comparison with Conventional Extraction Techniques

To evaluate the extraction efficiency of IFD by S-CO_2_ extraction, a comparison with conventional extraction methods was carried out. The dry fruits were preventively shredded before being processed. Also in this case, each methodology was evaluated in terms of extraction yield (%DW) and IFD recovery (%), calculated as reported in Section 4.5.

#### 4.6.1. Hydrodistillation

The hydrodistillation was conducted on 100 g of dry fruits following the methodology previously reported [4]. Specifically, the fruits were extracted with 1 L of distilled water (1/10 plant/solvent ratio) for 4 h, employing a Falc MA mantle (Falc Instruments, Treviglio, Italy) and a Clevenger apparatus for the collection of the essential oil.

#### 4.6.2. Soxhlet Extraction

The Soxhlet extraction was carried out with a Soxhlet Universal Extractor E-800 (Büchi Labortechnik AG, Flawil, Switzerland) on 20 g of dry fruits with 200 mL of *n*-hexane as the extracting solvent (plant/solvent ratio of 1/10). This solvent was selected accordingly to the high lipophilicity of IFD. The extraction time was 240 min. After the extraction, the solvent was removed at 40 °C under reduced pressure.

### 4.7. Statistical Analysis

Regression analysis and mean effect plots were generated by employing Minitab 18 statistical software. Concerning the comparison with extraction techniques, mean extraction yields (%) and IFD recovery (%) were analyzed by repeated measures and one-way analysis of variance (ANOVA) followed by Tukey’s test, which were performed by employing Minitab 18 statistical software. The asterisks represent the significance of Tukey’s test according to the following: *** *p* < 0.001, and ** 0.01 < *p* < 0.001.

## 5. Conclusions

The choice of an appropriate extraction technique is often a crucial step for the extraction of bioactive compounds from botanical sources, especially when these molecules are thermosensitive. This is the case of IFD, which is converted into its isomer curzerene at high temperatures. In this work, an optimization of S-CO_2_ extraction of IFD from *S. olusatrum* was carried out for the first time, demonstrating the effectiveness of the methodology in terms of time consumption and temperature control, thus avoiding the Cope rearrangement of IFD. The optimized process led to an extraction yield of 8.50% and to an IFD recovery of 0.94%. This study also demonstrated that S-CO_2_ extraction is significantly more efficient than hydrodistillation in terms of IFD recovery, while being comparable to a traditional solvent extraction as Soxhlet. Concerning the avoidance of IFD conversion into its isomer curzerene, S-CO_2_ extraction results were more effective than hydrodistillation and were comparable to Soxhlet extraction. Overall, this work demonstrated the high feasibility of S-CO_2_ extraction for obtaining IFD from *S. olusatrum* and furnished a green and sustainable process as an alternative to conventional hydrodistillation or Soxhlet extraction.

## Figures and Tables

**Table 1:** Extraction yield (%DW) and IFD recovery (%) in the preliminary trials.

Trial	Extraction Yield (%)	IFD Recovery (%)
P1	1.73	0.57
P2	1.71	0.66
P3	1.73	0.78
P4	2.09	0.70
P5	2.30	0.76
P6	1.24	0.59
P7	1.71	0.69
P8	2.10	0.77
P9	1.49	0.77

**Table 2:** Extraction yield (% dry weight, DW) and IFD recovery (%) in the optimization trials.

Trial	Extraction Yield (%)	IFD Recovery (%)
O10	5.64	0.58
O11	6.96	0.81
O12	8.52	0.96
O13	7.32	0.88

**Table 3:** Experimental conditions of the preliminary trials indicated as ‘P’.

Trial	Pressure (MPa)	Extraction Time (min)	Static Mode (%)
P1	30	60	100.0
P2	30	60	75.00
P3	30	60	66.67
P4	40	60	66.67
P5	50	60	66.67
P6	30	90	100.0
P7	50	45	66.67
P8	50	90	66.67
P9	30	60	66.67

The static mode (%) is the % of static time with respect to the total extraction time.

**Table 4:** Experimental conditions for the optimization of the extraction predictors. The trials are indicated with “O”.

Trial	Pressure (MPa)	Extraction Time (min)	Static Mode (%)
O10	50	60	45
O11	30	60	45
O12	50	60	25
O13	30	60	25

The static mode (%) is the % of static time with respect to the total extraction time.

## Data Availability

The original contributions presented in this study are included in the article/Supplementary Material. Further inquiries can be directed to the corresponding author.

## Supplementary Materials

The following supporting information can be downloaded at: https://www.mdpi.com/article/10.3390/plants15071099/s1, Section S1: Figure S1: Calibration curve of isofuranodiene (IFD) (A) and curzerene (B), Figure S2: Regression analysis for the extraction yield (%) results, Figure S3: Regression analysis for the isofuranodiene (IFD) recovery (%) results, Figure S4: Contour plot showing the influence of pressure and static mode on the extraction yield; Section S2: Choice of the temperature for the S-CO_2_ extraction of IFD. Reference [24] is included in the Supplementary Materials.

## Abbreviations

The following abbreviations are used in this manuscript:

S-CO_2_	Supercritical CO_2_
IFD	Isofuranodiene
DW	Dry weight
LOD	Limit of detection
LOQ	Limit of quantification
S/N	Signal-to-noise
RSD	Relative standard deviation
