# Supplementary material for: Advances in Isofuranodiene Extraction from Smyrnium olusatrum L.: Supercritical Carbon Dioxide Extraction
Source: Plants (Basel). 2026 Apr 3;15(7):1099. doi: 10.3390/plants15071099 (PMC13074484; doi:10.3390/plants15071099)
Supplement: Supplementary file 1 [file plants-15-01099-s001.zip › plants-4195264-supplementary.pdf]

Supplementary material for

Advances in isofuranodiene extraction from *Smyrnium olusatrum* L.: supercritical carbon dioxide extraction

Eleonora Spinozzi[a], Giada Trebaiocchi[a], Riccardo Petrelli[a], Francesco Di Monaco[a], Marco Cespi[a], Maggi Filippo[a]

[a]Chemistry Interdisciplinary Project (ChIP), School of Pharmacy, Via Madonna delle Carceri, University of Camerino, 62032 Camerino, Italy. eleonora.spinozzi@unicam.it, giada.trebaiocchi@unicam.it, riccardo.petrelli@unicam.it, francesco.dimonaco@studenti.unicam.it, marco.cespi@unicam.it, filippo.maggi@unicam.it

*Corresponding author: Eleonora Spinozzi, eleonora.spinozzi@unicam.it, Chemistry Interdisciplinary Project (ChIP), School of Pharmacy, Via Madonna delle Carceri, University of Camerino, 62032 Camerino, Italy.

Section S1 2
Section S2 6
References 6

Section S1

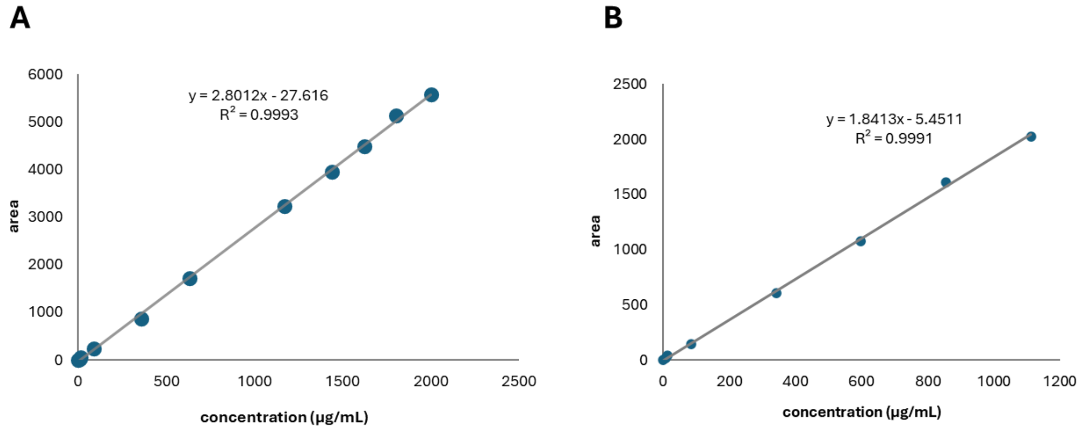

Figure S1. Calibration curve of isofuranodiene (IFD) (A) and curzerene (B).

Regression Analysis: Extraction yield (%) versus ... in); Static mode (%)

Backward Elimination of Terms

Candidate terms: Temperature (°C); Pressure (KPa); Time (min); Static mode (%)

	------Step 1------		------Step 2------		------Step 3------	
	Coef	P	Coef	P	Coef	P
Constant	6,84		6,97		5,97	
Temperature (°C)	-0,0338	0,484	-0,0346	0,456		
Pressure (KPa)	0,000068	0,016	0,000070	0,010	0,000063	0,009
Time (min)	0,0057	0,690				
Static mode (%)	-0,0366	0,042	-0,0338	0,030	-0,0375	0,011
S		0,615932		0,592189		0,582167
R-sq		78,59%		78,23%		77,05%
R-sq(adj)		70,03%		72,29%		73,22%
R-sq(pred)		*		*		62,62%
Mallows' Cp		5,00		3,17		1,72

α to remove = 0,05

Analysis of Variance

Source	DF	Adj SS	Adj MS	F-Value	P-Value
Regression	2	13,653	6,8263	20,14	0,000
Pressure (KPa)	1	3,251	3,2512	9,59	0,009
Static mode (%)	1	3,099	3,0986	9,14	0,011
Error	12	4,067	0,3389		
Lack-of-Fit	6	1,134	0,1890	0,39	0,864
Pure Error	6	2,933	0,4888		
Total	14	17,720			

Model Summary

S	R-sq	R-sq(adj)	R-sq(pred)
0,582167	77,05%	73,22%	62,62%

Coefficients

Term	Coef	SE Coef	T-Value	P-Value	VIF
Constant	5,97	1,50	3,97	0,002	
Pressure (KPa)	0,000063	0,000020	3,10	0,009	1,40
Static mode (%)	-0,0375	0,0124	-3,02	0,011	1,40

Regression Equation

Extraction yield (%) = 5,97 + 0,000063 Pressure (KPa) - 0,0375 Static mode (%)

Figure S2. Regression analysis for the extraction yield (%) results.

Regression Analysis: IFD Recovery (%) versus ... (min); Static mode (%)

Backward Elimination of Terms

Candidate terms: Temperature (°C); Pressure (KPa); Time (min); Static mode (%)

	-------Step 1-------		-------Step 2-------		-------Step 3-------	
	Coef	P	Coef	P	Coef	P
Constant	1,122		1,108		1,0250	
Temperature (°C)	-0,00187	0,577	-0,00225	0,395		
Pressure (KPa)	-0,000000	0,816				
Time (min)	0,001412	0,212	0,001369	0,166	0,001329	0,162
Static mode (%)	-0,00554	0,013	-0,005334	0,002	-0,005487	0,001
S		0,0369952		0,0333446		0,0329706
R-sq		89,22%		89,05%		87,15%
R-sq(adj)		78,43%		82,48%		82,87%
R-sq(pred)		*		*		77,81%
Mallows' Cp		5,00		3,06		1,77

	-------Step 4-------	
	Coef	P
Constant	1,0739	
Temperature (°C)		
Pressure (KPa)		
Time (min)		
Static mode (%)	-0,004989	0,001
S		0,0364226
R-sq		81,71%
R-sq(adj)		79,10%
R-sq(pred)		73,86%
Mallows' Cp		1,78

α to remove = 0,05

Analysis of Variance

Source	DF	Adj SS	Adj MS	F-Value	P-Value
Regression	1	0,041485	0,041485	31,27	0,001
Static mode (%)	1	0,041485	0,041485	31,27	0,001
Error	7	0,009286	0,001327		
Total	8	0,050771			

Model Summary

S	R-sq	R-sq(adj)	R-sq(pred)
0,0364226	81,71%	79,10%	73,86%

Coefficients

Term	Coef	SE Coef	T-Value	P-Value	VIF
Constant	1,0739	0,0680	15,79	0,000	
Static mode (%)	-0,004989	0,000892	-5,59	0,001	1,00

Regression Equation

IFD Recovery (%) = 1,0739 - 0,004989 Static mode (%)

Figure S3. Regression analysis for the isofuranodiene (IFD) recovery (%) results.

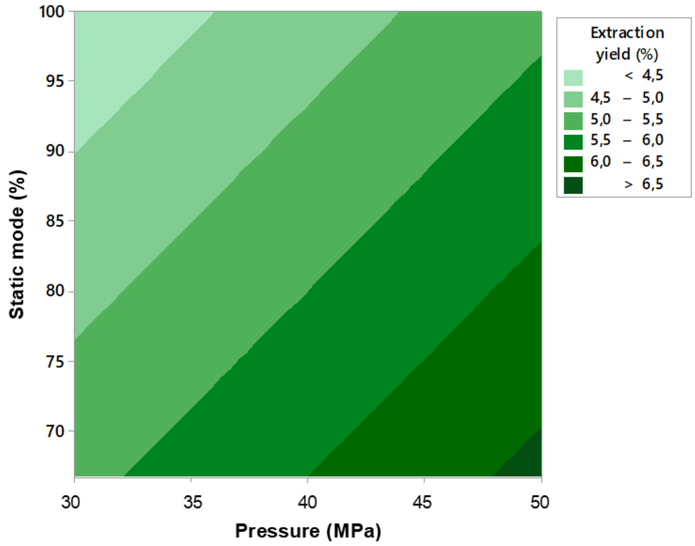

Figure S4. Contour plot showing the influence of pressure and static mode on the extraction yield.

Section S2

Choice of the temperature for the S-CO_2 extraction of IFD

The temperature of 45°C has been chosen accordingly to a study previously conducted in our laboratory and not yet published. In detail, we showed that the conversion of IFD into curzerene is time-independent up to 40 °C, whereas the conversion increases with longer times at higher temperatures. The choice not to screen lower temperatures than 45°C was driven by the fact that, generally, higher temperatures are reported to positively influence the supercritical CO_2 extraction efficiency by increasing solute vapour pressure and improving mass transfer [24]. For this reason, a temperature of 45°C was chosen as an optimal compromise to ensure the supercritical CO_2 conditions, to minimize the risk of Cope rearrangement, and to currently increase the extraction efficiency, thus remaining within the typical temperature range reported for thermolabile compounds (approximately 35-60 °C) [24].

References

24. Reverchon, E.; De Marco, I. Supercritical fluid extraction and fractionation of natural matter. J. Supercrit. Fluids **2006**, *38*(2), 146-166. https://doi.org/10.1016/j.supflu.2006.03.020
